# Aromaticity-driven laser photo-responses and binding efficiency in IAF-conjugated natural products for neurodegenerative disease targets

**DOI:** 10.1038/s41392-025-02560-w

**Published:** 2026-02-06

**Authors:** Nik Humaidi Nik Zulkarnine, Vahid Faramarzi, Michael Taeyoung Hwang

**Affiliations:** Department of BioNano Technology, Gachon University, Sujeong-Gu, 1342 Seongnam-Daero, Seongnam-si 13120 Republic of Korea

**Keywords:** Biophysics, Imaging

**Dear Editor**,

Alzheimer’s disease (AD) remains devastating neurodegenerative disorder with enormous societal and clinical impact, largely due to the challenge of detecting pathogenies changes early and the absence of effective disease-modifying treatments. Central to AD pathogenic is the dysregulated proteolytic processing of amyloid precursor protein (APP), primarily mediated by β-site APP cleaving enzyme (BACE1) which initiates the accumulation of amyloid-β (Αβ) peptides. The enzymes α-secretase (ADAM10) and the Aβ-degrading neprilysin are widely recognized as critical intervention points. Yet, there is a pressing need for probes that can both sensitively detect and modulate these targets in complex biological settings.^1,2^ Current imaging and therapeutic agents often lack both the optical responsiveness and biochemical selectivity needed for early AD intervention. Integrating optical sensing with target-specific binding, supported by in silico docking and structure-based screening, now offer rational path toward precision for theranostic molecular probe.^3^

Here, we selected two structurally divergent natural products as model scaffolds: oridonin, a rigid diterpenoid lacking extended π-conjugation and ammosamide B, a planar aromatic chromophore with distinct π-electron delocalization. Figure 1a illustrates the conceptual framework for capturing the photophysical responses of molecular probes. Both their native forms and immunoaffinity fluorescence (IAF)-tagged derivatives were analyzed through TDDFT simulations and docking studies. Photo-absorption spectra (Fig. 1b, “Photo-absorption spectra”) reveal that ammosamide B exhibits strong *π*– π^*^ transitions with high oscillator strengths, consistent with its extended aromatic framework. IAF tagging introduces additional conjugation, leading to bathochromic shifts and enhanced intensity. In contrast, oridonin shows weak absorption in its native state, attributed to its limited conjugation network; however, IAF conjugation introduces donor-acceptor character, significantly amplifying its optical signal and making its viable imaging candidate. Field enhancement contour maps (Fig. 1b, “Field enhancement contour maps”) further show localization of electrical field gradients. Ammosamide B derivative display continuous, spatially delocalized fields, whereas oridonin’s response becomes markedly enhanced only upon tagging. Exciton dynamic confirm that coherent exciton wave packets form rapidly and persist longer in ammosamide B systems, favoring strong signal transduction. We note that extending ${\rm{\pi }}$-system delocalization through conjugated tag system shows visualizes strong optical excitation responses after excitation and visualizing persistence of excited state distribution in a femtosecond period. HOMO-LUMO transition contribution analysis and FMO visualization corroborate this observation: ammosamide B maintains π-delocalization across entire scaffold, while oridonin exhibits more localized charge separation that is broadened and redistributed upon IAF tagging. Together, these results argue both inherent aromaticity and electronically coupled tag conjugation are critical in controlling optical performance. Temporal evolution exciton-dipole moment transition, HOMO-LUMO transition contribution map and FMO supporting these conclusions are presented in Extended Data (Available via Figshare).

**Fig. 1:**
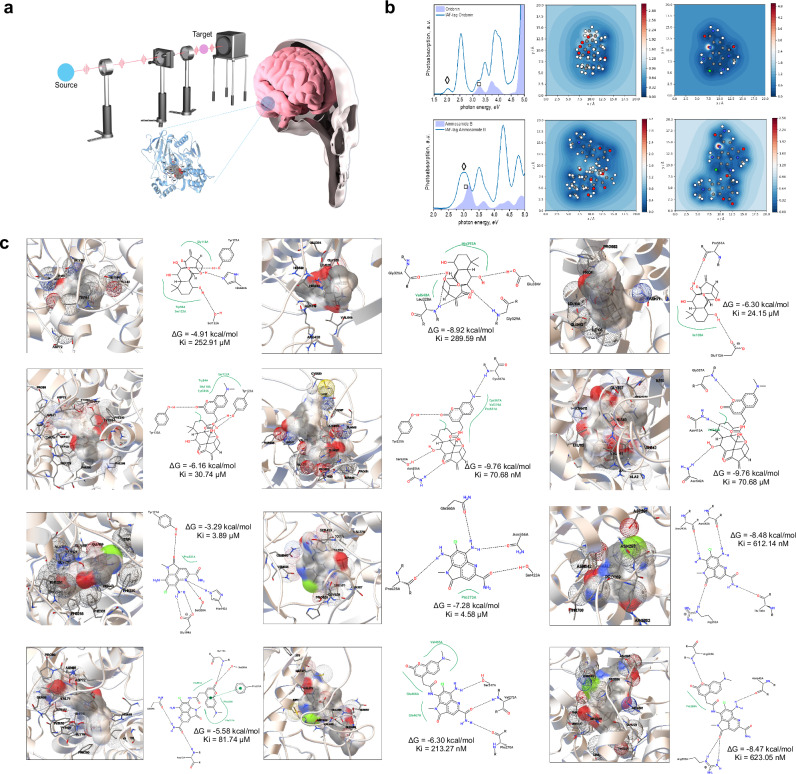
Computational evaluation of aromatic scaffold tuning for dual optical and targeting properties in Natural product-based fluorescent probes. **a** Conceptual schematic of laser-based excitation platform designed to measure photophysical responses of molecular probes, alongside of fluorescence-labeled natural product positioned within brain tissues indicating regions targeted for imaging or therapeutic intervention in neurodegenerative disease models. The synthesis of these compounds has been reported previously.^4^^,^^5^. **b** Comparative photo-absorption and excitonic properties of oridonin and ammosamide B with their derivatives. Photo-absorption spectra of oridonin and its derivatives (upper) and ammosamide B and its derivatives (lower). “▫” and “◊” represent the excitation peaks for each molecule. Field enhancement contour maps for oridonin (upper left) and its derivatives (lower left) and ammosamide B (right upper) and its derivatives (lower left). **c** Molecular docking interactions of natural products are depicted in rows as follows: oridonin (first row), IAF-tagged oridonin (second row), ammosamide B (third row), and IAF-tagged oridonin (fourth row). Each compound is shown docked with three target proteins organized in columns: BACE (left), ADAM10 (middle), and Neprilysin (right). For each ligand-protein pair, the left image displays the 3D structure of the docking complex, whereas the corresponding right image presents 2D interactions maps providing key molecular contacts, such as hydrogen bonds, hydrophobic interactions and *π*–*π* stacking

Docking analyses for BACE1 (Fig. 1c, left column), oridonin engages with well-defined hydrophobic pockets via van der Waals hydrogen-bonds, yielding potent inhibition (ΔG = −4.91 kcal/mol, Ki = 252.91 μM). Tagging with IAF, however compromises its steric bulk fit and altered orientation reduce affinity nearly tenfold (ΔG = −6.16 kcal/mol, Ki = 30.74 μM). In the docked pose, oridonin’s hydroxyl and carbonyl groups form directional hydrogen-bonds that anchor the ligand, while the diterpenoid framework sits in a hydrophobic cleft, producing a compact and well-seated complex. The loss of complementarity after the IAF-tagging arises from steric interference that perturbs these contacts and weakens dispersion stabilization. Ammosamide B, despite relatively shallow binding in its native state, improving docking energy with IAF through inhibition is paradoxically weakened by electrostatic mismatch and increased torsional strain, which can decouple docking scores from actual binding affinity. For ADAM10 (Fig. 1c, middle column), only IAF-conjugated oridonin achieves nanomolar affinity (ΔG = −9.76 kcal/mol, Ki = 70.68 nM), taking advantage of its scaffold’s adaptability to exploit complementarity in the enzyme’s groove. Here, the IAF-conjugated forms a multipoint hydrogen-bonding network and $\pi -\pi$ contacts with surrounding residues, resulting in deeply buried and electrostatically stabilized pose. Ammosamide B’s rigid planar geometry limits its optimal interactivity, and IAF tagging does not provide better binding performance. ADAM10 preferentially stabilizes IAF-oridonin complex because its combination of aromatic extension and conformational adaptability enables deeper insertion, additional hydrogen bonding and stronger dispersion coupling.

For Neprilysin (Fig. 1c, right column), Ammosamide B (ΔG ≈ −8.48 kcal/mol, Ki = 612.14 nM) and its IAF-derivative maintain stable binding, attributed to their planar, polar nature and preservation of key hydrogen-bond and dispersion interaction upon chemical modification. In docked pose, both ligands lie flat within catalytic cleft, forming multiple directional hydrogen-bonds and $\pi -\pi$ contacts that produce an electrostatically balanced, dispersion-stabilized complex. This planar arrangement maximizes charge delocalization and minimizes steric strain, therefore contributing to overall complex stability. Oridonin interacts weakly with Neprilysin (ΔG ≈ −6.30 kcal/mol, Ki = 24.15 μM) because of its rigid, polycyclic scaffold is sterically incompatible with the flat pocket, preventing deep insertion and extensive hydrophobic enclosure. However, the IAF-oridonin conjugate (ΔG ≈ −9.76 kcal/mol, Ki = 70.68 μM) shows markedly improved binding. The flexibility of IAF tag enable reorientation of the amine-carbonyl axis, optimizing hydrogen-bond geometry and stronger interaction dispersion with residues at the cleft entrance. It’s extended of $\pi$-surface in IAF enhances electronic polarizability and buried contact area, yielding more enthalpically stabilized, dispersion-dominated and geometrically complementary complex.

In conclusion, Ammosamide B is optically superior due to its intrinsic aromaticity and electronic delocalization, but its planar rigidity can limit target engagement in enzymes requiring adaptive fitting, such as ADAM10. Oridonin, while optically muted in its native form, is highly tunable: IAF conjugation transforms its photo-physics and enables selective, high-affinity binding to certain target protein. Aromaticity is not merely an optical property amplifier but also a determinant of molecular recognition. The degree and topology of aromatic π-electron delocalization establish structural continuum that connects molecular geometry with photophysical response, recognition selectivity and adaptive binding. In this view, controlling delocalization through rational scaffold modification offers a general principle for tuning optical activity and molecular targeting across chemically diverse system.

## Supplementary information

Supplementary Information

## Data Availability

All data supporting the findings of this study are available within the article or from the corresponding author upon reasonable request. High-resolution exciton-dynamics visualization, HOMO-LUMO transition maps, and frontier molecular orbital (FMO) figures are available at 10.6084/m9.figshare.30892604.
