# Supplementary material for: Aromaticity-driven laser photo-responses and binding efficiency in IAF-conjugated natural products for neurodegenerative disease targets
Source: Signal Transduct Target Ther. 2026 Feb 6;11:47. doi: 10.1038/s41392-025-02560-w (PMC12876877; doi:10.1038/s41392-025-02560-w)
Supplement: Supplementary file 1 — Supplementary Information [file 41392_2025_2560_MOESM1_ESM.docx]

Supplementary Materials for

**Aromaticity-driven Laser Photo-responses and Binding Efficiency in IAF-conjugated Natural Products for Neurodegenerative Disease Targets**

Nik Humaidi Nik Zulkarnine, Vahid Faramarzi* and Michael Taeyoung Hwang*

Department of BioNano Technology, Gachon University, 1342 Seongnam-Daero, Sujeong-Gu, Seongnam-si 13120, Republic of Korea

*Correspondence authors:

Dr. Vahid Faramarzi, Department of BioNano Technology, Gachon University, 1342 Seongnam-Daero, Sujeong-Gu, Seongnam-si 13120, Republic of Korea. *Email address:* vahid.fra @gmail.com

Dr Michael Taeyoung Hwang, Department of BioNano Technology, Gachon University, 1342 Seongnam-Daero, Sujeong-Gu, Seongnam-si 13120, Republic of Korea. *Email address:* anol81@gachon.ac.kr

**This Supplementary information includes:**

Theoretical and computational details.

Detailed results and discussion.

Supplementary Figure S1.

Supplementary Table S1.

**Theoretical and Computational details**

All TDDFT calculations were conducted using the GPAW code package,^1,2^ as previously reported for free-gaseous state organic molecules.^3,4^ To capture the ground-state properties of the natural product molecules of interest, let us consider the Kohn–Sham equations in the context of the Localized Combination Atomic Orbital (LCAO) basis function. The idea is to expand the Kohn–Sham orbitals I**(r)** with a set of atomic orbital χ_µ_**(r** – **R**_atom_) centered on each atom at which µ indicates the basis functions and **R**_atom_ is the atomic position.

| $Ф_{i}\left( \mathbf{r} \right)=\sum_{\mu}^{N_{\mathrm{basis}}} c_{\mu i}\chi_{\mu}(\mathbf{r}-\mathbf{R}_{\mathrm{atom}}\mathbf{)}$ | **EQ 1** |
| --- | --- |

where N_basis_ **_is_** the total number of basis functions. The Kohn–Sham equation is then rewritten into LCAO basis functions:

| $\hat{h}_{\mathrm{KS}} Ф_{i}\left( \mathbf{r} \right)= \epsilon_{i}Ф_{i}\left( \mathbf{r} \right)$ | **EQ 2** |
| --- | --- |

LCAO function **(2)** is then expanded into **(1)** and the resulting equation is multiplied with $\chi_{v}^{*}(\mathbf{r}-\mathbf{R}_{\mathrm{atom}}\mathbf{)}$ before integrating it over **r** to yield **EQ 3**.

| $\sum_{\mu}^{N_{\mathrm{basis}}} \left[ \hat{h}_{KS,\mu v}- \epsilon_{i}S_{\mu v} \right]c_{\mu i}= 0$ | **EQ 3** |
| --- | --- |

where $S_{\mu v}$is the overlapping matrix element described as follows:

| $S_{\mu v}= \int\chi_{\mu}^{*}\left( \mathbf{r}-\mathbf{R}_{\mathrm{atom}} \right)\chi_{v}\left( \mathbf{r}-\mathbf{R}_{\mathrm{atom}} \right)d\boldsymbol{r}$ | **EQ 4** |
| --- | --- |

Kohn–Sham matrix elements are defined as:

| $\hat{h}_{\mathrm{KS}} Ф_{i}\left( \mathbf{r} \right)= T_{\mu v} + V_{\mathrm{eff}, \mu v}$ | **EQ 5** |
| --- | --- |

$T_{\mu v}$ and $V_{\mathrm{eff}, \mu v}$ are the kinetic energy and effect potential matrix element defined as $\frac{1}{2}\int\chi_{\mu}^{*}\left( \mathbf{r}-\mathbf{R}_{\mathrm{atom}} \right){\nabla^{2}\chi}_{v}\left( \mathbf{r}-\mathbf{R}_{\mathrm{atom}} \right)d\boldsymbol{r}$ and $\int\chi_{\mu}^{*}\left( \mathbf{r}-\mathbf{R}_{\mathrm{atom}} \right){\nabla^{2}\chi}_{v}\left( \mathbf{r}-\mathbf{R}_{\mathrm{atom}} \right)d\boldsymbol{r}$, respectively. $c_{\mu i}$ and $\epsilon_{i}$ are the coefficients and eigenvalues, respectively, obtained by solving **EQ 3**. The linear response absorption was obtained by time evolution of electronic density (r, t) under the influence of an external time-dependent potential $V_{eff, \mu v}$ (r, t) shown as **EQ 1.**

| $i\frac{\partial}{\partial t}\psi_{i}\left( r,t \right)=\left[ -\frac{1}{2}\nabla^{2}+v_{ext}\left( r,t \right)+v_{xc}\left[ n;r,t \right]+v\left[ r,t \right]+u\left[ n;r,t \right] \right]\psi_{i}\left( r,t \right)$ | **EQ 6** |
| --- | --- |

The external potential $v_{ext}\left( r,t \right)$ includes interaction of electrons with an external field, i.e., explicitly both spatial (*r*) and time (*t*) dependent. The equation $v_{ext}\left( r,t \right)=V_{ext}\left( r \right)+A\left( t \right)cos(\omega t)$ consists of $V_{ext}\left( r \right)$, a static external potential, and A(t) is the amplitude of the laser pulse that varies with *t* and the angular frequency of laser, $\omega$. Initially, the geometry of the natural product molecule was centered, and geometric optimization was conducted using the conjugate gradient Broyden–Fletcher–Goldfarb–Shanno (BFGS) energy minimization algorithm until all forces were below 0.01 eV/angstrom. Real-time TDDFT calculations were performed by approximating the electrons with norm-conserving Troullier-Martin pseudopotentials, calculated within the LDA-PZ exchange-correlation function integrated into **EQ 6**. The pseudo-orbitals were represented on a real-space grid with a spacing of 0.2 Å in a simulation box. Ground-state calculations were configured for an unpolarized spin system. TDDFT calculation emphasizes the LCAO of the electronic wave function with a double-ζ polarized (dzp) basis set. ^5,6^$1\times{10}^{-3}$^5,6^Utilization of the PW91/LDA hybrid exchange-correlation function in the adiabatic limit, along with self-interaction correction, was chosen to address computational limitations over a more accurate exchange-correlation function such as B3LYP^5,6^. The linear absorption spectra of oridonin, and Ammosamide B, and their IAF-tagged derivatives were obtained by propagating the Kohn–Sham wave function orbital system kicked in 10 as a time step and propagating 3500 steps following the delta-peaked uniform electric field strength of $1\times{10}^{-3}$ au ultrafast laser pulse.^5,7^The same computational setup was used to characterize the HOMO-LUMO transition behavior and calculate the electron dynamics induced by the ultrafast laser pulse. 2D transition contribution maps (TCM) were used to analyze the electron-hole excitation contribution of FA_p_ in the photoabsorption spectra, satisfying the following equation.$\frac{1}{2\pi\sigma^{2}}$ $\frac{1}{2\pi\sigma^{2}}$^8,9^ 2D Gaussian function utilized, $\frac{1}{2\pi\sigma^{2}}$ and $\frac{1}{2\pi\sigma^{2}}exp\left[ \frac{{{(\varepsilon}_{0}{-\varepsilon}_{i})}^{2}+{{(\varepsilon}_{0}{-\varepsilon}_{a})}^{2}}{2\sigma^{2}} \right]$ for discretization of the *i* → *a* transition contribution

| $M_{\omega}^{TCM}\left( \varepsilon_{0}{,\varepsilon}_{u} \right)=\sum_{ia} \omega_{ia}(\omega)\frac{1}{2\pi\sigma^{2}}exp\left[ \frac{{{(\varepsilon}_{0}{-\varepsilon}_{i})}^{2}+{{(\varepsilon}_{0}{-\varepsilon}_{a})}^{2}}{2\sigma^{2}} \right]$ | **EQ 7** |
| --- | --- |

Next, the Z-orientation transition dipole moment, µ was captured through **EQ 8** derived from the two-level Hamiltonian system where $\left. |g \right\rangle$ and $\left. |e \right\rangle$ represent the ground state and excited stated, respectively.

| $\mu\left( t \right)= \left\langle\Psi(t) \vert\hat{\mathbf{d}}\Psi(t) \right\rangle=\left\vert c_{e}\left( t \right) \right\vert^{2}\left( \mu_{e}{-\mu}_{g} \right)+2R \left[ c_{g}^{*}(t)c_{e}(t)e^{i\left( E_{e}-E_{g} \right)t}\mathbf{d}_{\mathrm{ge}} \right]$ | **EQ 8** |
| --- | --- |

**Field Enhancement mapping via FD TDDFT.** Using the optimized geometries from previous calculations, the same computational setup was used to calculate the energy of these natural products in their ground states. The resonant frequencies from the first absorption peak, FAp, were selected to calculate the Fourier transform of the density oscillation and obtain the electric near field. Although the LCAO basis is efficient for computing excitation energies and their absorption spectra, lack of spatial completeness is required to resolve fine electrical field variations in and around natural products. Field enhancement regions typically extend into vacuum or dielectric surroundings, where LCAO basis functions are sparse or absent. Therefore, the FD mode was used to calculate polarization and local electric-field intensities with high spatial fidelity.

**Molecular Docking analysis.** To investigate the interactions between immunoaffinity-conjugated natural product probes, molecular docking was performed using AutoDock 4.2.^10^ The structures of BACE1 (PDB ID: 6ej3), ADAM10 (PDB ID: 6BE6), and neprilysin (PDB ID: 6SH2) were obtained from RSCB (<https://www.rcsb.org/>). All structures were preprocessed using Chimera by removing water molecules (HOH) and unwanted residues. Kollman and Gasteiger charges were then assigned, and polar hydrogen atoms were added. Optimized natural products, both with and without the immunoaffinity tag, were initially docked to the target structure using AutoDock Vina v1.1.2 using a blind docking approach.^11,12^ Optimized natural products, both with and without the immunoaffinity tag, were initially docked to the target structure using AutoDock Vina v1.1.2 using a blind docking approach.^11,12^ A grid box of 126 × 126 × 126 points with a spacing of 0.375 Å was used to cover the entire receptor surface and identify potential binding sites. Docking validation was performed using established method to obtain RMSD value between each docked trials.^13^ The binding configuration with the lowest predicted binding energy and the most favorable RMSD was selected for focused docking. Subsequent docking was performed using AutoDock 4.2 in a smaller grid box of 65 × 65 × 65 points at the same spacing (0.375 Å). The Lamarckian Genetic Algorithm (LGA) was applied for 200 runs to ensure thorough conformational sampling and accurate binding mode prediction. Proteins and docking sites were viewed using UCSF Chimera 1.173^14^ and Protein Plus (<https://proteins.plus/>).^15-18^

**Reference**

1 Walter, M. et al. Time-dependent density-functional theory in the projector augmented-wave method. The journal of chemical physics **128** (2008).

2 Mortensen, J. J., Hansen, L. B. & Jacobsen, K. W. Real-space grid implementation of the projector augmented wave method. Physical review B **71**, 035109 (2005).

3 Mokkath, J. H. Revealing the nature of localized and delocalized excitations of DNA. Journal of Physics and Chemistry of Solids **170**, 110960, doi:<https://doi.org/10.1016/j.jpcs.2022.110960> (2022).

4 Mokkath, J. H. Electric Near-field Modulations of Charged Deoxyribonucleic Acid Nucleobases. Plasmonics **15**, 1411-1420 (2020).

5 Toulouse, J. in Density Functional Theory: Modeling, Mathematical Analysis, Computational Methods, and Applications 1-90 (Springer, 2022).

6 Domagała, M. et al. Testing of exchange-correlation functionals of DFT for a reliable description of the electron density distribution in organic molecules. **23**, 14719 (2022).

7 Krumland, J., Valencia, A. M., Pittalis, S., Rozzi, C. A. & Cocchi, C. J. T. J. o. C. P. Understanding real-time time-dependent density-functional theory simulations of ultrafast laser-induced dynamics in organic molecules. **153** (2020).

8 Mokkath, J. H. Delocalized exciton formation in C60 linear molecular aggregates. Physical Chemistry Chemical Physics **23**, 21901-21912 (2021).

9 Rossi, T. P., Kuisma, M., Puska, M. J., Nieminen, R. M. & Erhart, P. Kohn–Sham decomposition in real-time time-dependent density-functional theory: An efficient tool for analyzing plasmonic excitations. Journal of Chemical Theory and Computation **13**, 4779-4790 (2017).

10 Morris, G. M. et al. AutoDock4 and AutoDockTools4: Automated docking with selective receptor flexibility. **30**, 2785-2791 (2009).

11 Eberhardt, J., Santos-Martins, D., Tillack, A. F., Forli, S. J. J. o. c. i. & modeling. AutoDock Vina 1.2. 0: New docking methods, expanded force field, and python bindings. **61**, 3891-3898 (2021).

12 Trott, O. & Olson, A. J. J. J. o. c. c. AutoDock Vina: improving the speed and accuracy of docking with a new scoring function, efficient optimization, and multithreading. **31**, 455-461 (2010).

13 Shivanika, C. et al. Molecular docking, validation, dynamics simulations, and pharmacokinetic prediction of natural compounds against the SARS-CoV-2 main-protease. 1 (2020).

14 Pettersen, E. F. et al. UCSF Chimera—a visualization system for exploratory research and analysis. Journal of computational chemistry **25**, 1605-1612 (2004).

15 Schöning-Stierand, K. et al. ProteinsPlus: a comprehensive collection of web-based molecular modeling tools. Nucleic Acids Research **50**, W611-W615, doi:10.1093/nar/gkac305 %J Nucleic Acids Research (2022).

16 Schöning-Stierand, K. et al. ProteinsPlus: interactive analysis of protein–ligand binding interfaces. Nucleic Acids Research **48**, W48-W53, doi:10.1093/nar/gkaa235 %J Nucleic Acids Research (2020).

17 Fährrolfes, R. et al. ProteinsPlus: a web portal for structure analysis of macromolecules. Nucleic Acids Research **45**, W337-W343, doi:10.1093/nar/gkx333 %J Nucleic Acids Research (2017).

18 Stierand, K. & Rarey, M. J. A. m. c. l. Drawing the PDB: protein− ligand complexes in two dimensions. **1**, 540-545 (2010).
